# OsHUS1 Facilitates Accurate Meiotic Recombination in Rice

**DOI:** 10.1371/journal.pgen.1004405

**Published:** 2014-06-05

**Authors:** Lixiao Che, Kejian Wang, Ding Tang, Qiaoquan Liu, Xiaojun Chen, Yafei Li, Qing Hu, Yi Shen, Hengxiu Yu, Minghong Gu, Zhukuan Cheng

**Affiliations:** 1 State Key Laboratory of Plant Genomics and Center for Plant Gene Research, Institute of Genetics and Developmental Biology, Chinese Academy of Sciences, Beijing, China; 2 Key Laboratory of Crop Genetics and Physiology of Jiangsu Province/Key Laboratory of Plant Functional Genomics of Ministry of Education, Yangzhou University, Yangzhou, China; INRA, France

## Abstract

Meiotic recombination normally takes place between allelic sequences on homologs. This process can also occur between non-allelic homologous sequences. Such ectopic interaction events can lead to chromosome rearrangements and are normally avoided. However, much remains unknown about how these ectopic interaction events are sensed and eliminated. In this study, using a screen in rice, we characterized a homolog of HUS1 and explored its function in meiotic recombination. In *Oshus1* mutants, in conjunction with nearly normal homologous pairing and synapsis, vigorous, aberrant ectopic interactions occurred between nonhomologous chromosomes, leading to multivalent formation and subsequent chromosome fragmentation. These ectopic interactions relied on programed meiotic double strand breaks and were formed in a manner independent of the OsMER3-mediated interference-sensitive crossover pathway. Although early homologous recombination events occurred normally, the number of interference-sensitive crossovers was reduced in the absence of OsHUS1. Together, our results indicate that OsHUS1 might be involved in regulating ectopic interactions during meiosis, probably by forming the canonical RAD9-RAD1-HUS1 (9-1-1) complex.

## Author Summary

Meiosis is a special type of cell division that generates gametes for sexual reproduction. During meiosis, recombination not only occurs between allelic sequences on homologs, but also between non-allelic homologous sequences at dispersed loci. Such ectopic recombination is the main cause of chromosomal alterations and accounts for numerous genomic disorders in humans. To ensure genomic integrity, those ectopic recombinations must be quickly resolved. Despite the importance of ectopic recombination suppression, the mechanism underlying this process still remains largely unknown. Here, using rice as a model system, we identified the rice HUS1 homolog, a member of the RAD9-RAD1-HUS1 (9-1-1) complex, and elucidated its roles in meiotic recombination. In *Oshus1*, vigorous ectopic interactions occur between nonhomologous chromosomes, and the number of crossovers is reduced. We suspect that OsHUS1 participates in regulating ectopic interactions during meiosis, probably by forming the canonical RAD9-RAD1-HUS1 (9-1-1) complex.

## Introduction

Meiosis is a highly dynamic process in which chromosomes undergo dramatic structural changes and movements [1],[2]. During the course of meiosis, intimate interactions develop between homologous chromosomes. Among these interactions, homologous recombination (HR) and pairing are the core events that occur during the production of functional gametes [3], [4]. Meiotic recombination is a powerful determinant that creates genetic diversity and provides mechanical stability for the accurate separation of homologous chromosomes. Therefore, meiotic recombination has a strong bias towards homologous chromosomes rather than sister chromatids and is mediated by a complex mechanism [5], [6]. After DNA replication in the premeiotic S phase, a proteinaceous axis is assembled between two chromatids. Homologous recombination then occurs along the chromosomes, beginning with the formation of programmed double strand breaks (DSBs). In conjunction with the initiation of recombination, homologous chromosomes begin to align in pairs.

Studies have shown that for most species, homologous pairing depends on homologous recombination [7]. However, recombination not only occurs between allelic DNA sequences on homologs, but it also frequently occurs between dispersed non-allelic DNA segments that share high sequence similarity [8]. The latter recombination pattern is usually referred to as ectopic recombination (ER, also known as non-allelic homologous recombination). As eukaryotic genomes are rich in repeated DNA sequences, ER can produce chromosomal rearrangements, which in humans result in numerous genomic disorders [9], [10]. Despite the adverse impact of ER on genome integrity, ER occurs relatively frequently; in budding yeast, the frequency of ER is roughly on par with that of allelic recombination [11], [12]. To avoid the deleterious consequences of ER, cells have evolved multiple strategies to suppress ER formation [13]. One strategy is preventing DSB formation in or near DNA repeats. In budding yeast (*Saccharomyces cerevisiae*), suppression of DSBs in rDNA repeats depends strongly on silent information regulator 2 (Sir2), which encodes a histone deacetylase that promotes the formation of a closed, compact chromatin structure in the rDNA and other regions. Sir2 may suppress DSBs in rDNA in part through the formation of a nucleosomal conformation that is not permissive for SPO11 activity [14]. The second strategy is preventing the use of non-allelic homologous templates for recombination and/or favoring the use of allelic templates. In budding yeast, homologous alignment and synapsis restrict the ability of ectopically located sequences to find each other and recombine [15]. There are also reports on the competition between normal allelic recombination and ER [16]. As both mechanisms involve preventing ectopic interaction intermediate formation, we classified these events as ectopic interaction preventing mechanisms in this study. The frequent occurrence of ER in yeast suggests that these mechanisms cannot totally prevent all ER initiation [17]–[19]. Once a non-allelic partner is used as the template and ER intermediates are built, a mechanism monitors and resolves those ER intermediates into non-crossovers. This ER-eliminating mechanism can be classified as a surveillance mechanism. Studies in *S. cerevisiae* have shown that the mismatch repair proteins Pms1 and Msh2 are likely to be involved in this mechanism, although direct evidence for this is still lacking. Several DNA helicases, including Sgs1 in yeast and BLM in humans, may also possess anti-crossover activities that are potentially involved in preventing deleterious outcomes of meiotic ER [13].

Rad9, Hus1, and Rad1 (in *S. cerevisiae*: Ddc1, Mec3, and Rad17) interact in a heterotrimeric complex (dubbed the 9-1-1 complex), which resembles a PCNA-like sliding clamp [20], [21]. In response to genotoxic damage, the toroidal 9-1-1 complex is loaded around damage sites, collaborating with ATM and ATR to carry out its best known function of activating the DNA damage checkpoint [22]. In addition, studies have revealed functional interactions between 9-1-1 and multiple partners, most notably translation polymerases, base excision repair enzymes, and mismatch repair factors [23], [24]. This evidence implies that 9-1-1 also plays a direct role in DNA repair. In addition to its role in the conventional somatic DNA damage response, 9-1-1 also plays roles in meiosis. In *S. cerevisiae*, it is evident that Ddc1 colocalizes with Rad51 on meiotic chromosomes and is required for the pachytene checkpoint [25]. The *rad17* mutants exhibit aberrant synapsis and increased rates of ER during meiosis [26]. In mouse, RAD1 was found to be associated with both synapsed and unsynapsed chromosomes during prophase I [27]. Recently, the HUS1 homologs in *Drosophila* and mouse were reported to be essential for meiotic DSB repair [28], [29].

The function of the 9-1-1 complex in suppressing meiotic ER was first suggested in yeast [26]. However, this function has not been reported in higher organisms, likely due to a lack of direct cytological evidence. In this study, we aimed to isolate genes that are involved in ectopic interaction suppression. Several mutants showing normal homologous pairing at pachytene and the appearance of ectopic interactions at diakinesis were isolated. Among these, two allelic mutants were characterized in detail. These mutants were found to be mutated in the functional homolog of fission yeast and mammalian *HUS1*. In the *Oshus1* mutants, meiotic homologous pairing took place normally during prophase I, while nonhomologous chromosomes interacted vigorously as well. Multivalents were frequently found to be arranged on the equatorial plate at metaphase I. Chromosome bridges and fragments occurred at anaphase I and telophase I, rendering the mutants completely sterile. These results suggest that OsHUS1 might specifically function in sensing and removing aberrant associations between non-allelic sequences during meiosis, probably via the 9-1-1 complex.

## Results

### Cloning of *OsHUS1*

Among our rice sterile mutant libraries, 16 lines with phenotypes meeting the criteria mentioned above were isolated. One of the mutant lines, *S7678*, which was derived from Nipponbare (a *japonica* cultivar) tissue culture, was selected for further study. Based on information about its mutation (see below), the mutant was named *Oshus1-1*. The *Oshus1-1* plants did not exhibit defects in vegetative growth under natural growth conditions, except for total male sterility (Figure S1). Fertile plants and sterile plants from the progeny of *Oshus1-1^+/−^* produced a 3∶1 segregation ratio (fertile, 214; sterile, 66), which established this mutant as a single recessive mutant (χ^2^ = 0.30; P>0.05). When we pollinated the mutant flowers with wild-type pollen, the mutant did not set seed, indicating that female fertility is also affected in this mutant.

We isolated *OsHUS1* by map-based cloning. A mapping population was constructed by crossing *Oshus1-1^+/−^* plants to Nanjing 11 (an *indica* cultivar) plants. The mutant gene locus was mapped to a physical region of approximately 100 kb on the long arm of chromosome 4. According to information obtained from the public database (Rice Genome Annotation Project, http://rice.plantbiology.msu.edu), we sequenced several genes in this region. As a result, a point mutation (A to T) was found in the gene *Os04g44620*, which introduced a stop codon (AAG to TAG) in the second exon. We named the mutant *Oshus1-1* based on the homology of the protein sequence.

Next, we isolated another mutant from Huanghuazhan (an *indica* cultivar), which has the same phenotype as that of *Oshus1-1*. Using map-based cloning and DNA sequencing, we found that this mutant carries a ten-nucleotide deletion in the fourth exon of *OsHUS1*, causing frame shift and premature stop codon formation. We named this allele *Oshus1-2*. The chromosome behavior in *Oshus1-2* meiocytes was the same as *Oshus1-1* (Figure S2A).

We generated a gene-specific p35S *OsHUS1*-RNAi construct and used it to transform Yandao 8 (a *japonica* cultivar) rice. Most *OsHUS1*-RNAi lines showed a severe reduction in fertility (93%, n = 30), and the chromosome behavior in the male meiocytes of these lines mirrored that of *Oshus1* (Figure S2B). From these results, we conclude that the mutation in the *OsHUS1* gene led to the sterility phenotype.

### Characterization of *OsHUS1*

There are three full-length cDNA sequences of *Os04g44620* published in the Rice Genome Annotation Project website, including AK107445, AK101159, and AK064120. Using RT-PCR and RACE (rapid amplification of cDNA ends) on young panicles, we found that AK064120 is the correct sequence for this gene. Alignment of the cDNA sequence with the genomic sequence revealed that *OsHUS1* is composed of six exons and five introns (Figure S3). The open reading frame of *OsHUS1* has a length of 981 bp, encoding a 326 amino-acid peptide. Using BLASTp, we found that OsHUS1 shares some similarity (approximately 25% identity and 45% similarity) with the HUS1 protein in mammals and fission yeast (Figure S4). Reciprocal BLAST searches further confirmed that the isolated protein is the closest relative of HUS1 in rice (Figure S5).

As shown above, there were several defects during meiosis in *Oshus1*. We then examined the spatial and temporal expression patterns of *OsHUS1*. Using quantitative RT-PCR, we found that *OsHUS1* could be detected as early as the seedling stage. In adult-stage rice, *OsHUS1* was expressed not only in young panicles but also in vegetative organs such as leaves, roots, and internodes (Figure S6), with the highest expression observed in leaf blades.

### Chromosome behavior in *Oshus1-1*

**Figure 1:**
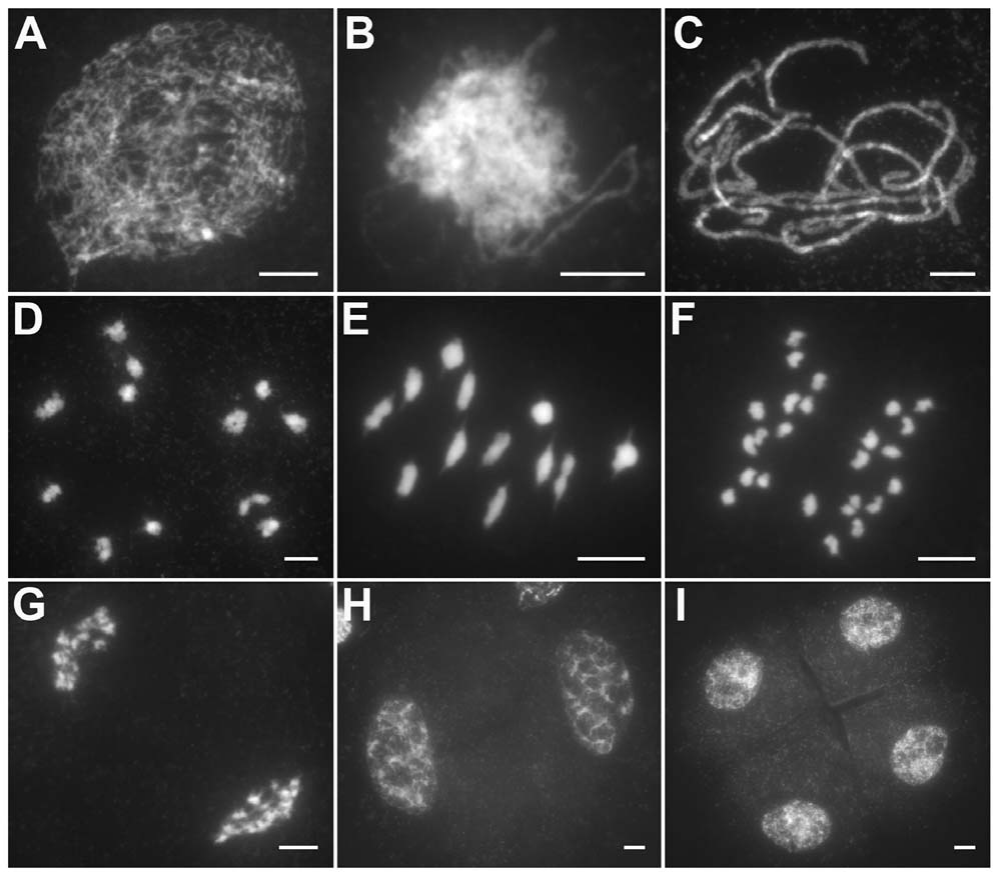
Meiosis in the wild type. (A) Leptotene. (B) Zygotene. (C) Pachytene. (D) Diakinesis. (E) Metaphase I. (F) Anaphase I. (G) Telophase I. (H) Dyad. (I) Tetrad. Bars, 5 µm.

The behavior of meiotic chromosomes was revealed by 4′6-diamidino-2-phenylindole (DAPI) staining. In wild-type pollen mother cells (PMCs), meiosis began with chromosome condensation and the appearance of chromosomes as thin, thread-like structures at leptotene (Figure 1A). As zygotene progressing, homologous chromosomes underwent pairing and synapsis (Figure 1B). During pachytene, homologous pairing culminated in the formation of synaptonemal complexes (SCs; Figure 1C). After the disassembly of the SC at diplotene, the resulting 12 bivalents were further condensed, revealing the presence of chiasmata at diakinesis (Figure 1D). At metaphase I, the bivalents were aligned in the middle of the cell (Figure 1E). Homologous chromosomes separated and migrated toward opposite poles at anaphase I and telophase I (Figure 1F and 1G), generating dyads at the end of meiosis I (Figure 1H). Then, the dyads underwent meiosis II and finally produced tetrads (Figure 1I).

**Figure 2:**
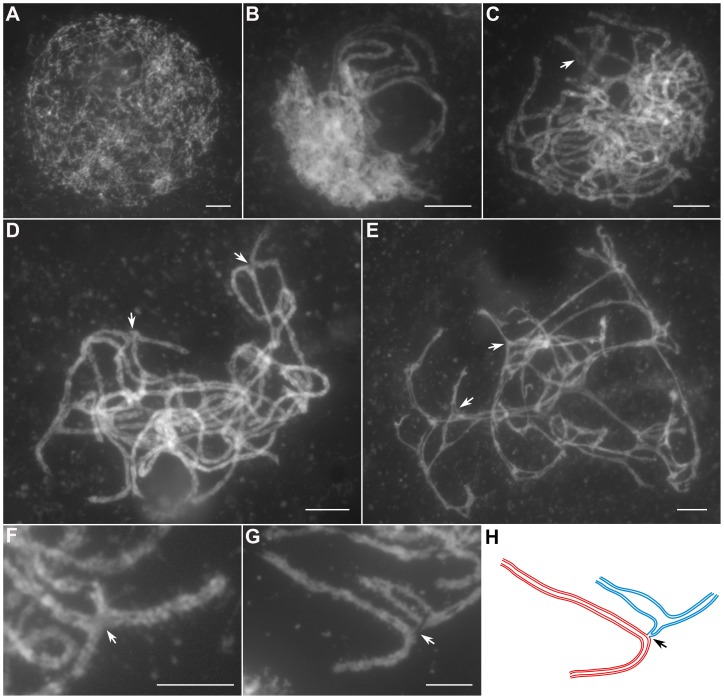
Meiotic chromosome behaviors of PMCs in the *Oshus1-1* mutant. (A) Leptotene. (B) Zygotene. (C) Early pachytene. (D) Middle pachytene. (E) Late pachytene. (F, G) The enlarged chromosome ectopic association regions. (H) Diagram shows chromosome configuration in (G). Red and blue lines indicate sister chromatids in different bivalents. The arrow indicates “bubble-like” region in (C) and ectopic association site in (D–H). Bars, 5 µm.

**Figure 3:**
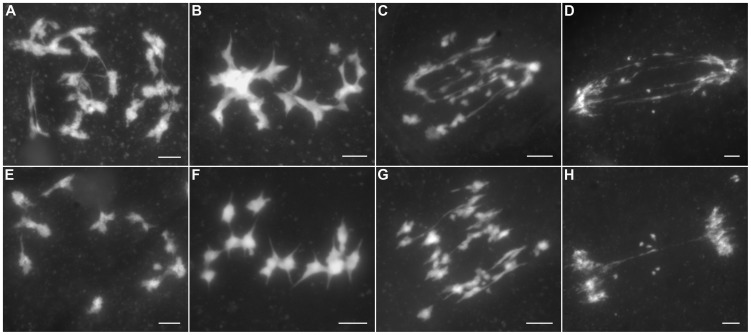
The aberrant chromosomal interactions in *Oshus1-1* resulted in multivalent associations and chromosome fragments. (A–D) exhibit PMCs with high frequencies of ectopic chromosomal interactions, while (E–H) show PMCs with low levels of ER. (A, E) Diakinesis. (B, F) Metaphase I. (C, G) Anaphase I. (D, H) Telophase I. Bars, 5 µm.

In *Oshus1-1* PMCs, the chromosome behaviors appeared the same as those observed in wild-type from leptotene to zygotene (Figure 2A and 2B). However, anomalies began to be manifested at early pachytene. At first glance, almost all homologous chromosomes aligned well. However, upon careful examination, we found that some regions of the chromosomes could not complete close alignment perfectly and exhibited “bubble-like” structures (Figure 2C). During middle pachytene stage, associations between nonhomologous chromosome were observed in all PMCs (n = 252), which caused the chromosomes to stick to each other (Figure 2D). At late pachytene, this type of association became more prominent (Figure 2E–H). At diakinesis, multivalents were detected in all PMCs (n = 521). These multivalents ranged in size from associations of four chromosomes to the extreme case of 24 chromosomes (Figure 3A, 3E); the average number of bivalents per cell was only 1.6. At metaphase I, multivalents and bivalents were located on the equatorial plate due to the drag force exerted on centromeres by spindle fibers (Figure 3B, 3F). During anaphase I, the multivalents and bivalents fell apart, and extensive chromosome bridges and fragments were observed (Figure 3C, 3G). At telophase I, two masses of chromosomes arrived at opposite poles of the nuclei, and several distinct dot-like chromosome fragments still remained on the equatorial plate (Figure 3D, 3H). In a few cells (4%, n = 881), up to 10 or 11 homolog pairs could be individualized at diakinesis and metaphase I. We also found some cells with a small amount of chromosome bridges and fragments at anaphase I and telophase I (Figure 3E, F). These types of defects were maintained during meiosis II, and no normal tetrad was produced.

### Synapsis is incomplete in most *Oshus1-1* meiocytes

**Figure 4:**
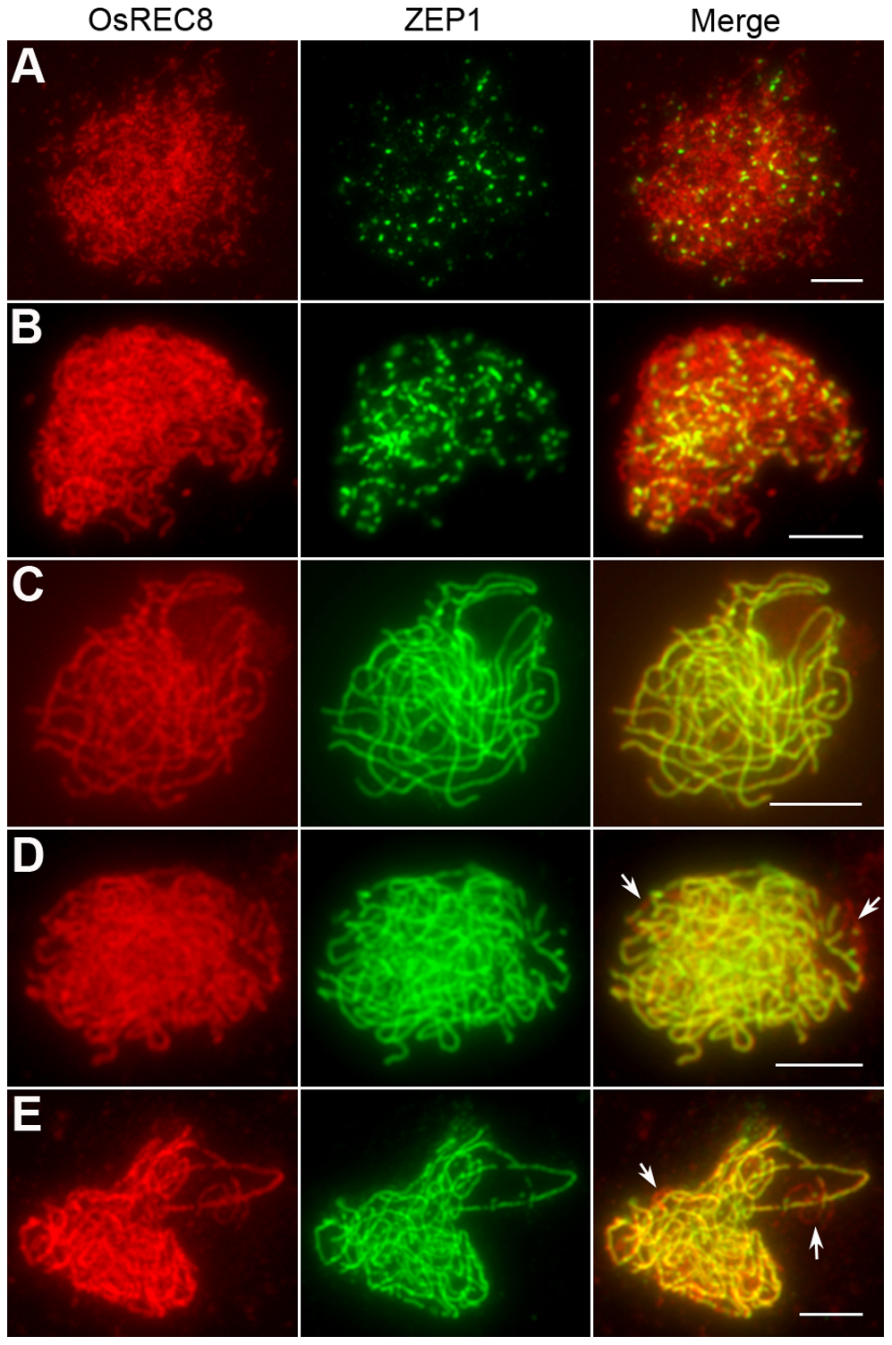
Immunolocalization of ZEP1 in the *Oshus1-1* mutant. (A) Leptotene. (B) Zygotene. (C–E) Pachytene. Image (C) shows complete ZEP1 signals along the meiotic chromosomes in the pachytene PMC, while (D) and (E) exhibit discontinuities of ZEP1 linear signals. Arrows indicate these gaps. Bars, 5 µm.

By performing DAPI staining of pachytene chromosomes, we found that in *Oshus1-1*, homologous chromosomes could pair normally. To validate whether normal SCs were affected by the mutation of *OsHUS1*, we performed immunofluorescent examination using antibodies against ZEP1 in *Oshus1-1* PMCs. ZEP1 is the transverse filament protein of SC in rice and hence, a perfect tool to mark the course of synapsis [30]. In leptotenic and zygotenic *Oshus1-1* PMCs, the ZEP1 patterns appeared as dots and short fragments, which were identical to those of the wild-type (Figure 4A and 4B). During pachytene, only approximately 10% (n = 300) of the meiocytes showed full-length ZEP1 signals along the homologous chromosomes (Figure 4C). In the remaining 90% of meiocytes, linear ZEP1 signals extended and could be detected along almost the entire chromosomes, with the exception of a few discontinuities/gaps, some of which exhibited the “bubble-like” structures mentioned above (Figure 4D and 4E). The discontinuities/gaps of ZEP1 signals indicate that the SC integrity might be slightly affected by the mutation of *OsHUS1*.

### *Oshus1* mutants show a reduced number of bright HEI10 foci

Many incidents during meiosis are believed to be interdependent, e.g., pairing is recombination-dependent in mammals and higher plants. The nearly normal SC assembly observed in *Oshus1* meiocytes is reminiscent of the proper loading of important factors involved in SC assembling and homologous recombination. To further verify the relationship between OsHUS1 and several other meiotic recombination factors, immunodetection was carried out in *Oshus1-1* using antibodies against PAIR3, PAIR2, OsZIP4, OsMER3, and HEI10.

**Figure 5:**
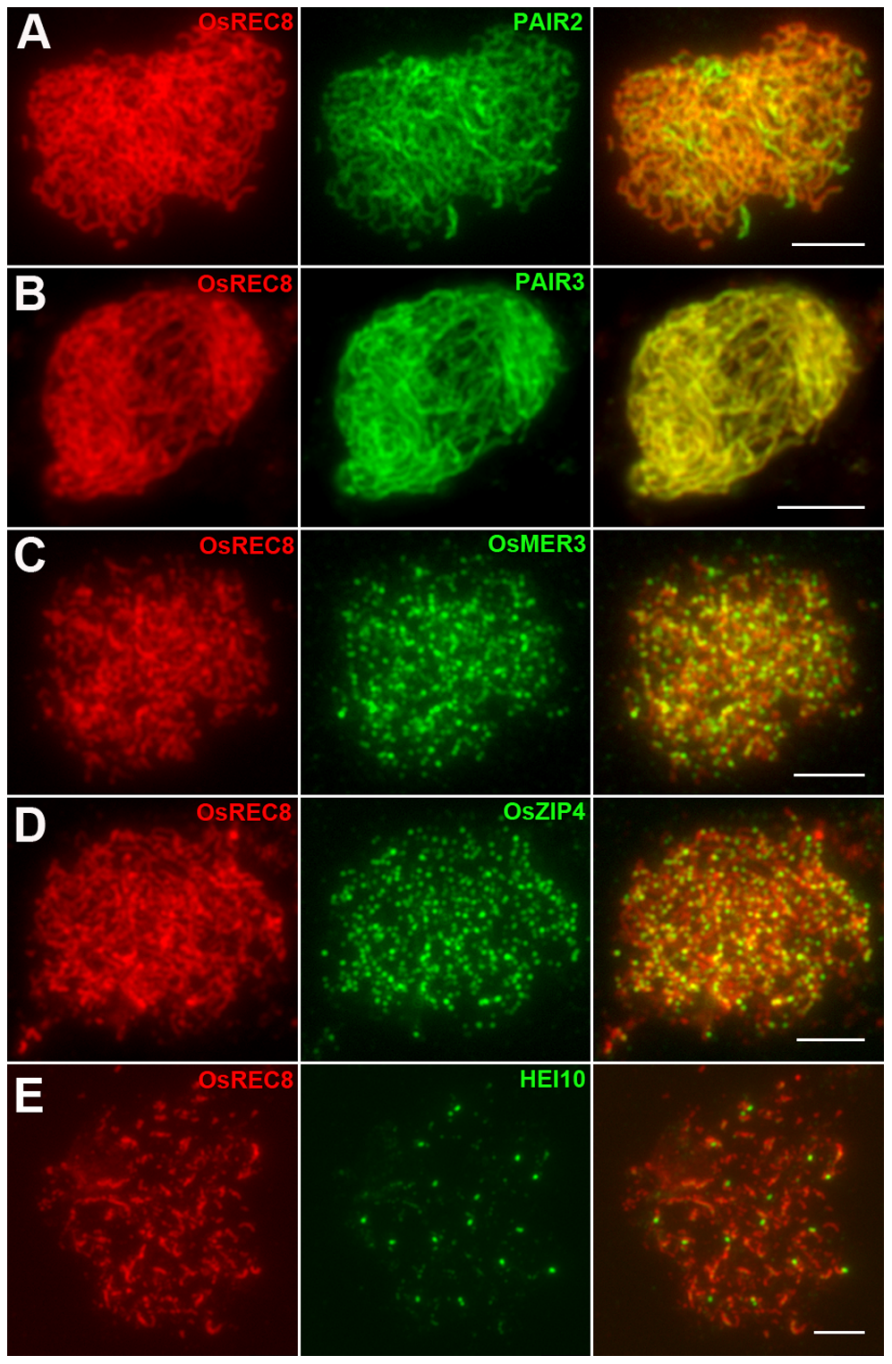
Immunolocalization of several meiotic elements in the *Oshus1-1* mutant. (A) PAIR2 signals at zygotene. (B) PAIR3 signals at zygotene. (C) OsMER3 signals at leptotene. (D) OsZIP4 signals at leptotene. (E) HEI10 bright foci at diplotene. Scale bars, 5 µm.

PAIR2 is the rice homolog of yeast HOP1 and *Arabidopsis* ASY1, which associates with unpaired chromosome axes at early meiosis I. PAIR3 is also an axis-associated protein that can bind both unpaired chromosomes and paired chromosomes. Both PAIR2 and PAIR3 are usually utilized to mark the meiotic chromosome axis, and they also play fundamental roles in the recombination process [31]–[33]. OsMER3 and OsZIP4 are members of the ZMM protein family and are essential for early meiotic HR in rice [34], [35]. In the *Oshus1-1* mutant, PAIR2 appeared as foci at leptotene and associated with the chromosome axis as linear signals at early zygotene (Figure 5A). PAIR3 signals were first observed as dots at early leptotene and then elongated gradually along the entire lengths of the chromosomes during zygotene (Figure 5B). The appearance of OsMER3 and OsZIP4 commenced at early leptotene, and the number of OsMER3 foci (average 257±15, n = 44, range 221–281) and OsZIP4 foci (average 299±22, n = 35, range 289–328) reached its peak at early zygotene (Figure 5C and 5D); similar results were obtained in the wild-type. At pachytene, both OsMER3 and OsZIP4 decreased rapidly and no signals were found in the later stages in the wild-type and *Oshus1-1*. The normal loading patterns of these four meiotic factors showed that early HR in *Oshus1-1* is not disturbed.

Previous studies suggest that the interference-sensitive pathway accounts for most of the crossovers (COs) in rice [34]–[36]. We thus wanted to know whether interference-sensitive COs were affected by the mutation of OsHUS1. The HEI10 prominent foci correspond to the interference-sensitive CO sites in rice [36]. We counted the number of HEI10 foci (average 16.9±1.9, n = 17, range 13–20) in *Oshus1-1* (Figure 5E) and compared that with the corresponding data for the wild-type (average 24.5±1.8, n = 30, range 22–28). We found that the mean number of HEI10 bright foci of *Oshus1-1* was significantly reduced compared with that of the wild-type (*t*_[45]_ = 13.8, *P*<0.01). Therefore, the number of interference-sensitive COs is reduced in *Oshus1-1* due to the loss of OsHUS1.

### Ectopic associations in *Oshus1-1* are dependent on PAIR1 but independent of OsRAD51C

**Figure 6:**
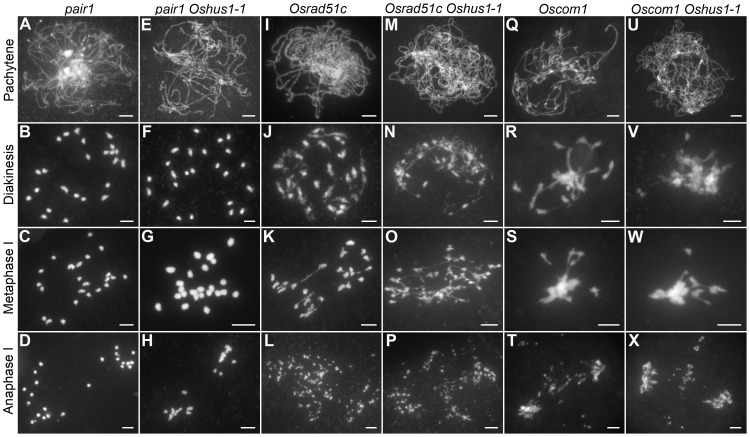
DAPI staining of *Oshus1-1* with other meiotic mutants. (A–D) *pair1*. (E–H) The *pair1 Oshus1-1* double mutant showing similar chromosome behaviors to those of the *pair1* single mutant. (I–L) *Osrad51c*. (M–P) The *Osrad51c Oshus1-1* double mutant displaying a cumulative effect of the two mutations. (Q–T) *Oscom1*. (U–X) The *Oscom1 Oshus1-1* double mutant showing similar chromosome behaviors to those of the *Oscom1* single mutant. Bars, 5 µm.

Meiotic recombination is initiated by the formation of DSBs, which is catalyzed by SPO11 proteins; these proteins have been identified in budding yeast, *Arabidopsis*, and animals [37]. However, to date, no *spo11* mutants have been isolated in rice [38]. Recently, three new proteins that are also implicated in DSB formation were reported in *Arabidopsis*, i.e., PRD1, PRD2, and PRD3 [39], [40]. Among these, PRD3 is thought to be the homolog of rice PAIR1. Furthermore, the phenotype of the *pair1* mutant (asynaptic, with no bivalent formation) is reminiscent of the phenotype observed in a mutant lacking DSBs [41]. We isolated an asynaptic mutant (Figure 6A–D), and it was proven to be a new allele of *pair1*. Then, *pair1 Oshus1-1* double mutants were generated using this new *pair1* allele. The double mutants showed a typical *pair1* phenotype, i.e., an absence of bivalents and lack of chromosome fragments at anaphase I (Figure 6E–H). Therefore, ectopic interactions, as well as chromosome fragmentations in *Oshus1-1*, require the formation of DSBs.

To learn whether OsHUS1 is involved in DSB repair pathway in rice meiosis, we generated *Osrad51c Oshus1-1* double mutants. OsRAD51C, like its functional homolog AtRAD51C, is essential for meiotic DSB repair [42]–[44]. In the *Osrad51c* mutant, homologous pairing and synapsis were defective at zygotene and pachytene, and univalents were observed at diakinesis and metaphase I (Figure 6I–K). In anaphase I, all of the univalents broke into fragments without any chromosome associations and scattered randomly in the nucleus (Figure 6L). These defects are consistent with the role of *Osrad51c* in meiotic DSB repair. In the *Osrad51c Oshus1-1* double mutant, a cumulative effect of the two single mutations was detected; homologous pairing was disrupted, and ectopic chromosome associations were detected in all meiocytes observed. (Figure 6M–O; n = 322). At anaphase I, extensive chromosome fragments were also produced (Figure 6P). Therefore, the occurrence of ectopic interactions in *Osrad51c Oshus1-1* suggests that ectopic interactions between nonhomologous chromosomes do not require OsRad51C.

OsCOM1 functions both in promoting homologous recombination and in resolving chromosome entanglements [45]. In the *Oscom1* mutant, both homologous pairing and synapsis were abolished at pachytene (Figure 6Q), and aberrant nonhomologous associations were detected. From diakinesis to metaphase I, the most obvious phenotype was an entangled chromosome mass (Figure 6R, S). At anaphase I, chromosome fragments were generated (Figure 6T). We also generated *Oscom1 Oshus1-1* double mutants. The phenotype of the *Oscom1 Oshus1-1* double mutant could not be distinguished from that of the *Oscom1* single mutant (Figure 6U–X), suggesting that OsHUS1 might function after OsCOM1 during meiosis. Of course, we cannot exclude the possibility that the ectopic interaction phenotype of *Oshus1* might be hidden by the severe chromosome entanglement of *Oscom1*.

### Ectopic associations in *Oshus1-1* are independent of OsMER3 and ZEP1

**Figure 7:**
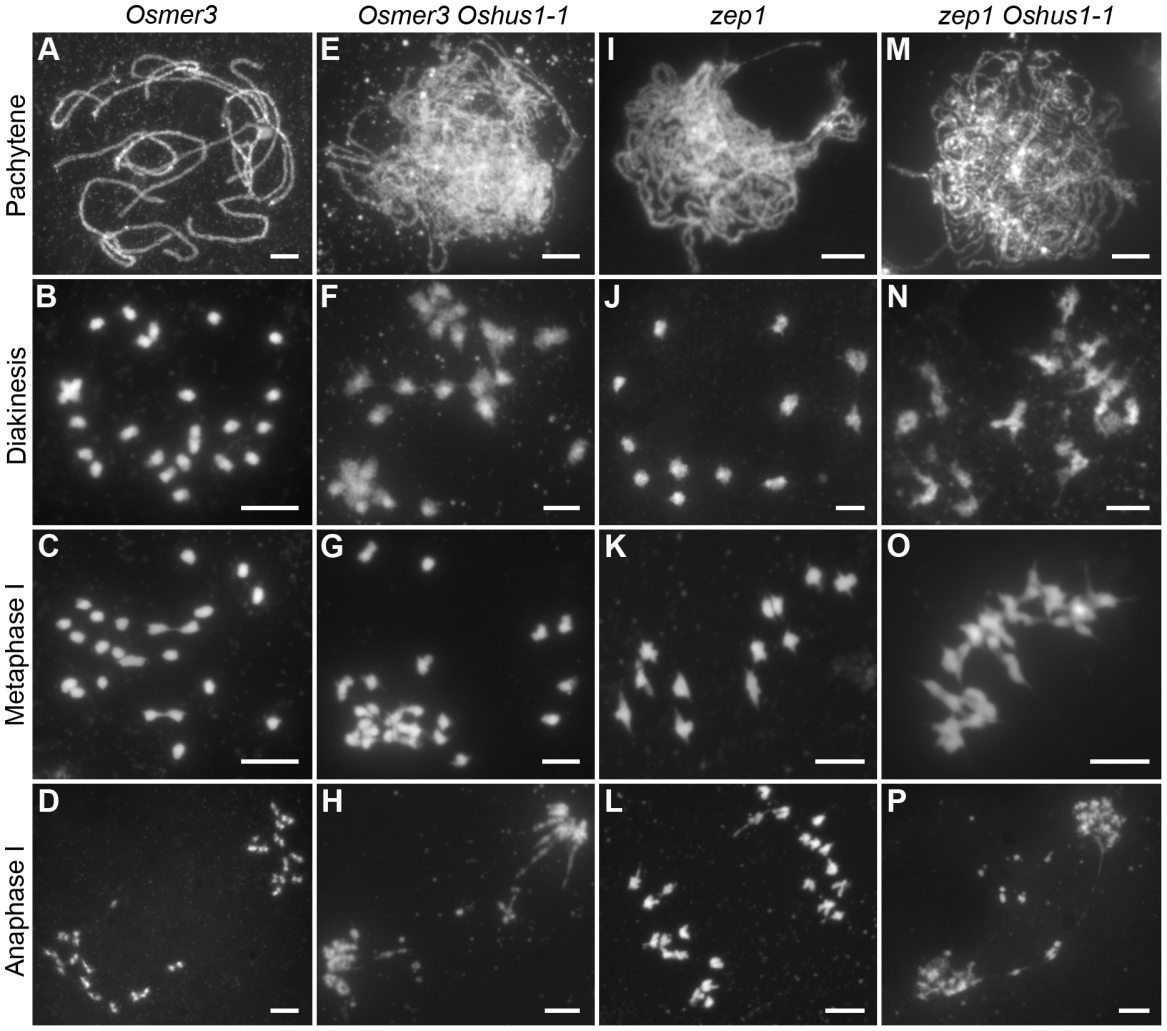
DAPI staining of *Osmer3, Osmer3 Oshus1-1*, *zep1* and *zep1 Oshus1-1* mutant. (A–D) *Osmer3*. (E–H) Homologous pairing is disrupted while ectopic associations are remained in *Osmer3 Oshus1-1*. (I–L) *zep1*. (M–P) The *zep1 Oshus1-1* double mutant exhibiting occurrence of ectopic association. Bars, 5 µm.

Since most COs in rice are derived from the interference-sensitive pathway, we set out to study the relationship between ectopic interactions and interference-sensitive COs. To this aim, the *Osmer3 Oshus1-1* double mutant was generated, and its chromosome behaviors were investigated. In *Osmer3*, fully aligned chromosomes were detected during pachytene (Figure 7A), indicating the homologous pairing is not affected by the mutation of *OsMER3*. However, during diakinesis and metaphase I, the mutant cells showed a mixture of both univalent and bivalent chromosomes (Figure 7B, C). In anaphase I, the bivalents separated normally but the scattered univalents segregated randomly (Figure 7D). Intriguingly, in the *Osmer3 Oshus1-1* mutant, homologous pairing was not observed at pachytene stage (Figure 7E). FISH experiments further confirmed that homologous pairing was disrupted in *Osmer3 Oshus1-1* meiocytes (n = 101, Figure S7). In diakinesis and metaphase I, both multivalents with ectopic interactions and univalents were detected in all meiocytes (n = 122, Figure 7F, G). The multivalents contained an average of 7.0 associated chromosomes (ranging from 2 to 22); the average number of univalents per cell was 8.2 (ranging from 0 to 16). At anaphase I, both univalents and multivalents were pulled toward two poles of the nucleus. Additionally, chromosome bridges and fragments were also found at this stage (Figure 7H). These results suggest that ectopic interactions in *Oshus1* arise independently from the OsMER3-mediated pathway.

To determine whether the defects in *Oshus1* are affected by synapsis, we also generated the *zep1 Oshus1-1* double mutant. In the *zep1* mutant, synapsis was totally disrupted, but 12 bivalents were present at metaphase I and segregated normally at anaphase I (Figure 7I–L). In the *zep1 Oshus1-1* double mutant, homologous chromosomes aligned along the entire length of the chromosome, but the SC was not assembled (Figure 7M). However, ectopic interactions were still clearly observed in all meiocytes (n = 298, Figure 7N–P). These results indicate that ectopic interactions are likely independent of synapsis in *Oshus1-1*.

### OsHUS1 localizes to meiotic chromosomes during early prophase I

**Figure 8:**
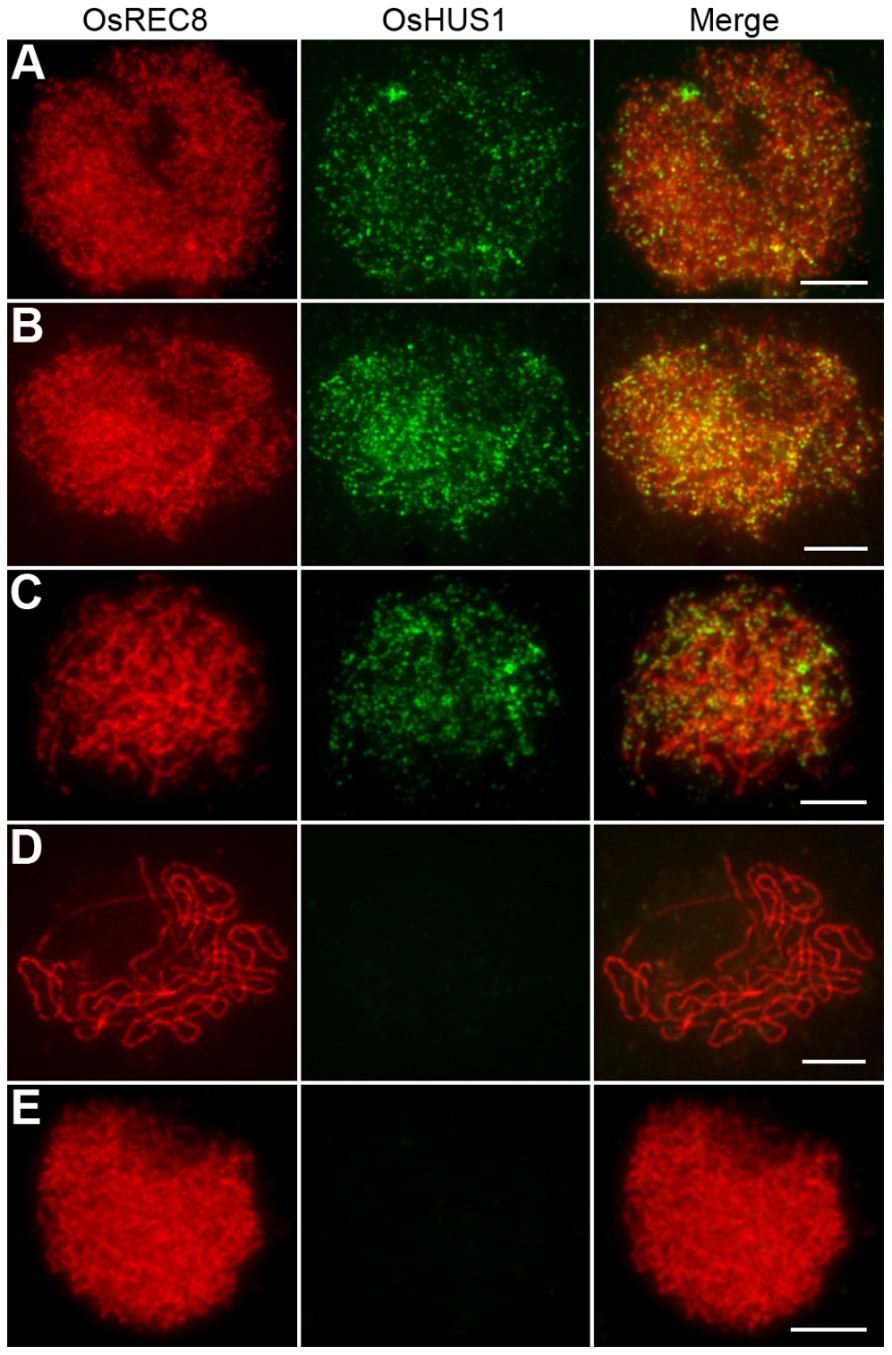
OsHUS1 localizes on meiotic chromosomes. (A) Leptotene. (B) Zygotene. (C) Early pachytene. (D) Late pachytene. (E) No signal was detected in *Oshus1-1* meiocytes at zygotene. Bars, 5 µm.

To further elucidate the role of OsHUS1 in meiosis, we prepared polyclonal antibodies in mice against the entire length of recombinant, His-tagged OsHUS1. Using antibodies against OsREC8 and OsHUS1, we performed dual immunofluorescence staining in rice PMCs. OsREC8, the cohesin protein in rice, was used to indicate the meiotic chromosome axes in this study [34], [46]. During leptotene, OsHUS1 proteins appeared as discrete foci in the nuclei and were loaded on the chromosome axes, as indicated by their full colocalization with OsREC8 (Figure 8A). The intensity of OsHUS1 then reached its peak at early zygotene, but this protein still appeared as foci rather than short lines (Figure 8B). At late zygotene, the number of OsHUS1 foci decreased, and many of them fell off the chromosomes (Figure 8C). At pachytene, the OsHUS1 immunostaining signal was completely absent in the nuclei (Figure 8D). No OsHUS1 signal was observed in male meiocytes of *Oshus1-1*, which confirmed the specificity of the OsHUS1 antibody (Figure 8E).

To further investigate the function of OsHUS1 protein, the immunolocalization pattern of OsHUS1 was investigated in *Osmer3*, *zep1*, and *pair1* mutants. The localization pattern of OsHUS1 was not obviously affected in *Osmer3* or *zep1* (Figure S8A, B). This result is consistent with the observation that no ectopic interaction was found in either of the mutants. On the contrary, in the *pair1* mutant, we failed to detect any OsHUS1 signals (Figure S8C), implying that the function of OsHUS1 depends on the formation of DSBs.

### *Oshus1-1* seedlings are hypersensitive to mitomycin C

**Figure 9:**
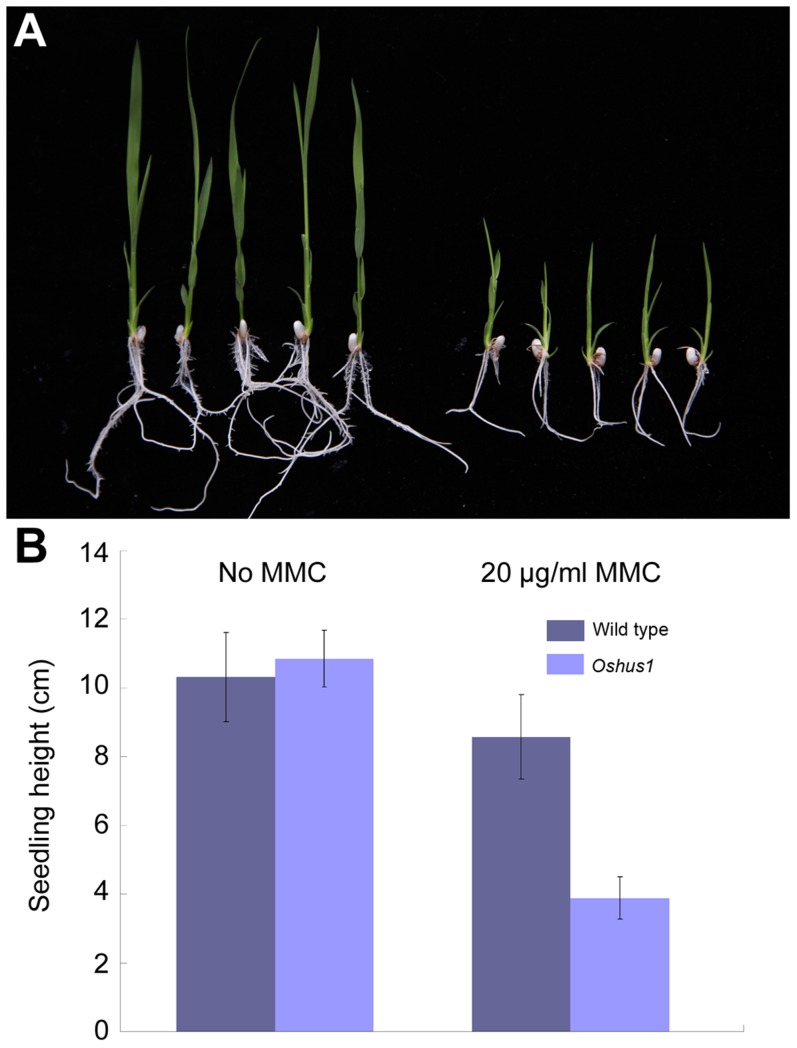
*Oshus1-1* is hypersensitive to MMC. (A) *Oshus1-1* seedlings (right) exhibited more growth retardation than that of wild-type seedlings (left) on 1/2 MS medium with MMC. (B) Statistical analysis of WT and *Oshus1-1* plant height.

In yeast and mammals, HUS1 protein is implicated in various DNA damage response pathways [47]–[51]. In rice, *OsHUS1* has the highest expression abundance in leaves, suggesting that this protein, like its counterparts in yeast and mammals, is potentially involved in the mitotic DNA damage response. To address this possibility, we tested whether *Oshus1* plants showed higher sensitivity to mitomycin C (MMC), a DNA cross-link agent, than wild-type plants. Surface-sterilized seeds from wild-type and *Oshus1-1* ^+/−^ plants were sown on solid 1/2 MS medium containing 0 or 20 µg/ml MMC. When planted on medium lacking MMC, the development of wild-type seedlings was identical to that of progeny derived from an *Oshus1-1* ^+/−^ plant. However, when treated with MMC, the development of wild-type seedlings was only slightly suppressed, while approximately one-quarter of the progeny derived from the *Oshus1-1* ^+/−^ plants showed severe growth retardation (Figure 9). Using a PCR genotyping assay, we determined that all of the severely growth-retarded seedlings were *Oshus1-1* ^−/−^ (n = 20). These data demonstrate that *Oshus1-1* rice is hypersensitive to MMC, indicating that OsHUS1 plays an important role in somatic DNA damage repair.

## Discussion

### OsHUS1 is involved in somatic DNA damage responses

HUS1 is thought to form a PCNA-like complex with its two partners, RAD9 and RAD1 [23]. HUS1 has been intensively investigated in yeast and mammals, with studies primarily focusing on the mitotic DNA damage response. A mutation in MEC3 (the HUS1 counterpart in budding yeast) results in delayed entry into the S phase and slow DNA replication in response to DNA damage-inducing agents [52]. Fission yeast lacking HUS1 also fails to arrest the cell cycle after DNA damage or the blocking of DNA synthesis [53]. Targeted disruption of mouse HUS1 causes embryonic lethality due to the accumulation of chromosome breaks [49].

In this study, we found that rice *hus1* seedlings were hypersensitive to the genotoxin MMC, suggesting that OsHUS1 has a conserved function in somatic DNA repair. Expression data for *OsHUS1* show high accumulation of its transcript in somatic tissues, which further supports the somatic role of this protein. These findings are also in agreement with the hypothesis that OsHUS1 in rice is the functional homolog of fungal and animal HUS1. By performing a BLASTp search, we found that the homologs of *S. pombe* RAD9 and RAD1 also exist in rice. In addition, RAD9 is also involved in the regulation of DNA damage repair in the model plant *Arabidopsis* [54]. Therefore, it is highly possible that OsHUS1 in rice, like its yeast and animal counterparts, also participates in somatic DNA repair responses by forming the 9-1-1 complex.

### OsHUS1 may be required for the suppression of ectopic interactions during meiosis

Studies in yeast and humans have revealed parallels between meiotic ER and allelic recombination, such as the observation that both processes occur during prophase I and are initiated by programmed DSBs. ER also results in crossover formation, which can affect genome stability during gametogenesis [12], [13], [55]. Therefore, ER should be inhibited, and/or its intermediates must be quickly eliminated, to ensure accurate homolog segregation during meiosis.

The function of the 9-1-1 complex in suppressing ER was first suggested in yeast [26]. However, to our knowledge, this function has not been reported in higher organisms, likely due to the lack of cytological evidence. Here, in *Oshus1* meiocytes, we noticed that at late pachytene, one homolog pair frequently adhered or fused to another homolog pair at several sites, forming cross-like shapes. At the pachytene to diplotene transition (in which homologous pairs began to separate partially due to SC disassembly), the associations became more pronounced. The most remarkable defects observed in *Oshus1* meiosis were multivalents at metaphase I and subsequent chromosome fragmentation.

The chromosome behaviors observed in the *pair1 Oshus1-1* double mutant indicate that ectopic interactions rely on meiotic DSBs in *Oshus1-1*, which supports the notion that ectopic and allelic interactions share a common mechanism [56]. DSB formation is essential for homologous chromosome pairing in meiosis [3]. Here, although strong ectopic interactions occurred in *Oshus1*, homologous pairing took place normally. The nearly perfect ZEP1 signals along the entire lengths of chromosomes at pachytene indicated that synapsis was not severely disturbed in *Oshus1*. In addition, OsZIP4 and OsMER3 localized normally in *Oshus1*. It is likely that the early ectopic intermediate-preventing system may function well, and excessive ectopic interaction initiations are prevented in a timely manner in *Oshus1*. Intriguingly, unlike the *Oshus1* and *Osmer3* single mutants, the *Oshus1 Osmer3* double mutant exhibited disrupted homologous pairing. In light of the competition between allelic and ectopic recombination [57] and the important roles they play during homologous pairing [3], it is attractive to consider that the increase in ectopic interactions and the decrease in allelic associations reduce the chance of homolog recognition and subsequent homolog alignment. Since homolog alignment mainly occurs at zygotene stage, it is reasonable to postulate that ectopic interactions initiate during or prior to zygotene. This hypothesis is consistent with the view that in yeast, ectopic recombination occurs concurrently with allelic recombination during meiosis [13].

Studies in budding yeast have revealed that ER occurs frequently during meiosis [11], [12]. However, it remains unknown whether ER also occurs frequently during plant meiosis. Here, we observed that all meiocytes showed the presence of multivalents in *Oshus1*. We therefore propose that in wild-type meiocytes, early ectopic interactions, accompanied by allelic interactions, may inevitably occur during homolog searching and homolog recognition. Once homolog recognition is accomplished, those ectopic interaction intermediates might be quickly detected and resolved by the surveillance mechanism. OsHUS1 is likely to be an important component of the surveillance mechanism that specifically eliminates ectopic interaction intermediates during meiosis.

### Ectopic associations are independent of the interference-sensitive CO pathway

In budding yeast, a physical assay revealed that levels of ER increase from 1% in wild-type to 3–5% in *rad17*, *rad24*, and *mec1-1* single mutants. HR is also reduced approximately two-fold in these mutants, from 25–30% in wild-type to 15% in *rad17*, *rad24*, and *mec1-1*. These data indicate that the increase in ER does not quantitatively account for the decrease in HR. Therefore, ER and HR likely occur via different pathways [26]. Here, we demonstrated that the loss of OsMER3 function did not affect ectopic interactions (through characterization of *Osmer3 Oshus1-1*), implying that these ectopic interactions do not arise from the interference-sensitive crossover formation pathway. In this study, we also observed that the average number of bright HEI10 foci was reduced in the *Oshus1-1* mutant, showing that the number of interference-sensitive COs was reduced in the absence of OsHUS1. Thus, the similar alterations in ectopic and allelic interactions between yeast and rice imply that the function of HUS1 may be conserved among different organisms.

Interference plays a role in both controlling and constraining the final distribution of COs. Although the mechanism underlying these processes remains unclear, it has been postulated that spreading interference signals are transmitted along the length of the chromosome axes [58]. Therefore, one possible explanation for the decrease in interference-sensitive CO number is that the spreading interference signals may also be transmitted through associated nonhomologous chromosome axes in *Oshus1*. Alternatively, it is possible that partial allelic interactions are redirected into ectopic interactions or resolved toward sister chromatids in the absence of OsHUS1.

### Possible functions of OsHUS1 during meiosis

Studies in yeast and mammals have shown that the 9-1-1 complex is involved in multiple DNA repair courses by binding to numerous partners, including base excision repair proteins and mismatch repair proteins [23]. Among these, the mismatch repair protein MSH2 is postulated to be involved in the intermediate elimination of ER [13]. In yeast and humans, MSH2-MSH6 heterodimer (MutSα) and MSH2-MSH3 heterodimer (MutSβ) are mismatch recognition factors that function in the mismatch repair pathway. Recent studies have revealed that each subunit of the 9-1-1 complex can interact with both the MSH2/MSH3 and MSH2/MSH6 complexes. In addition, the 9-1-1 complex can also stimulate the DNA binding activity of MutSα [59]. The biochemical properties of the 9-1-1 complex are likely similar during mitosis and meiosis. We therefore postulate that OsHUS1 may also function as a component of the 9-1-1 complex to sense ectopic interaction and further recruit MutS to eliminate ectopic interaction intermediates. The characterization of RAD9, RAD1, and MSH2 homologs in rice will deepen our understanding of the ER-eliminating mechanism.

## Materials and Methods

### Plant materials

*Oshus1-1* was derived from Nipponbare (a *japonica* cultivar) induced by tissue culture. *Oshus1-2* was derived from Huanghuazhan (an *indica* cultivar) induced by ^6^°Co∼γ ray radiation. The new *pair1* mutant allele was obtained from Nipponbare through tissue culture. In this allele, a *Tos17* retrotransposon was inserted in the 7^th^ exon of *PAIR1*. The new *Osrad51c* allele was derived from an *indica* rice variety Zhongxian 3037, induced by ^6^°Co∼γ ray radiation and found to have a premature stop codon in the 9^th^ exon of *OsRAD51C*. The *Oscom1* and *zep1* alleles employed in this study is *Oscom1*-*3* and *zep1-1*, respectively [30], [45]. Nipponbare was used as the wild type in the related experiments.

### Molecular cloning of *OsHUS1*

STS markers were developed based on sequence differences between *japonica* variety Nipponbare and *indica* variety 9311, which were used for map-based cloning of *OsHUS1*. Primers sequences were listed in Supporting information, Table S1. The cDNA sequence for *OsHUS1* was verified by 3′RACE. Total RNA was extracted from rice young panicles (6–8 cm) using TRIZOL reagent (Invitrogen). A measure of 3 µg RNA was reverse-transcribed with Oligo-Adaptor primer (CTGATCTAGAGGTACCGGATCC-d(T)16) using the superscript III RNaseH reverse transcriptase (Invitrogen). Two rounds of PCRs were carried out using Adaptor primer (CTGATCTAGAGGTACCGGATCC), gene specific primers RACE1F (TGTACCTTCTATGGTATTTC) and RACE2F (CTAGACTGACGGACAAGTCC). The product was cloned into pMD19-T vector (TaKaRa) and sequenced.

### Generating *OsHUS1-* RNAi transgenic plants

A 261bp fragment from the exons of *OsHUS1* was amplified by PCR with the primer pair OsHUS1RNAiF (AAGGATCCCTGACAGTAGCTGTTACTC) and OsHUS1RNAiR (AGGTCGACACCATAGAAGGTACAGTCGG). The product was introduced into the *Bam*HI-*Sal*I and *Bg* II-*Xho*I sites of the pUCCRNAi vector in an inverted repeat orientation. The stem-loop fragment was finally cloned into the pCAMBIA 1300 vector. The *OsHUS1-*RNAi construct was introduced into *Agrobacterium tumefaciens* strain EHA105 and transformed the *japonica* cultivar Yandao 8.

### Quantitative RT-PCR analysis

Total RNA was extracted from the internode, leaf, root, panicle and seedling of Nipponbare, and was reverse-transcribed into cDNA. Quantitative RT-PCR analysis was performed using the CFX96 Real Time system (Bio-Rad) and Eva Green (Biotium). The primer pair OsHUS1RTF (CTTGGTGTTCGTGCAACC) and OsHUS1RTR (ACCACCAGGAGAAATACC) was used. The standard control *UBIQUITIN* gene was examined with the primers UBI-RTF (CAAGATGATCTGCCGCAAATGC) and UBI-RTR (TTTAACCAGTCCATGAACCCG).

### Sensitivity test

Husked seeds from the wild-type plants and the heterozygous *Oshus1* ^+/−^ plants were surface sterilized. Then they were sown on solid 1/2 MS medium containing 20 µg/ml MMC (Solarbio) in a light incubator. Genotype and phenotype assays of the seedlings were assayed 14 days later.

### Antibodies

To generate the antibody against OsHUS1, the coding region of it was amplified from Nipponbare leaf cDNA with primer pair OsHUS1PETF (ATGGATCCATGAAGTTCAAGGCCTTC) and OsHUS1PETR (ATCTCGAGACTGCCAGGGTCAAGGAC), and then ligated to the *Bam*HI-*Xho*I site of the expression vector pET-30a (Novagen). The expression vector was transformed into *Escherichia coli* strain BL21 (DE3) and was induced for 3 h at 37°C by addition of 0.3 mM IPTG. His-tagged OsHUS1 were accumulated in the inclusion bodies and they were washed and subjected to SDS-PAGE. The main band of His-tagged OsHUS1 on the gel was cut off and powdered and used as an antigen against mice. The OsREC8, PAIR2, PAIR3, OsMER3, OsZIP4, HEI10, and ZEP1 polyclonal antibodies were used as described before [30], [32], [34], [35].

### Cytology

Young panicles of at meiosis stage were harvested and fixed in Carnoy's solution (ethanol:glacial acetic acid = 3∶1) for chromosome spreading. Meiotic chromosome preparation and immunofluorescence were performed as previously described [34]. The FISH procedure was performed as described [60]. Microscopy was conducted using a ZEISS A2 fluorescence microscope with a microCCD camera. Image capture and analysis was carried out using IPLab software (BD Biosciences).

## Supporting Information

Figure S1 Phenotype of the *Oshus1-1* mutant. (A) A wild-type plant; (B) A *Oshus1-1* plant; (C) Comparison of a wild-type (left) and a *Oshus1-1* panicle (right); (D, E) I_2_-KI staining of pollen grains in the wild type (D) and *Oshus1-1* mutant (E). Bars, 50 µm.
(TIF)

Figure S2 Meiotic chromosomes at Metaphase I in *Oshus1-2* and *OSHUS1* RNAi plant. (A) *Oshus1-2*. (B) An *OSHUS1* RNAi line. Scale bars, 5 µm.
(TIF)

Figure S3 Structure of the *OsHUS1* gene. Exons are represented by black boxes. Gray boxes show the untranslated regions. The position of the *Oshus1* mutation is indicated by an arrow.
(TIF)

Figure S4 Alignment of HUS1 homologues. Identical amino acids are shaded in black whereas similar amino acids are shaded in gray.
(TIF)

Figure S5 Phylogenetic tree of the 20 homologs defined by OsHUS1. The tree is constructed using MEGA 4.0 based on the neighbor-joining method. Numbers next to branches are clade credibility values.
(TIF)

Figure S6 Relative expression level of *OsHUS1* in different tissues analyzed by quantitative RT-PCR. Values are means ±SEM (standard error of mean) of three independent experiments and value of panicle is set as 1.
(TIF)

Figure S7 Detection of homologous chromosome pairing revealed by FISH in *Oshus1-1, Osmer3* and *Osmer3 Oshus1-1*. (A–C) Pachytene; (D) Diakinesis; FISH signals of 5S rDNA are in green, signals of the BAC clone a0065A15 on the long arm of chromosome 9 are in red, and chromosomes are in blue stained with DAPI. Bars, 5 µm.
(TIF)

Figure S8 Dual immunolocalization of OsREC8 and OsHUS1 in *Osmer3*, *zep1* and *pair1* PMCs. (A) *Osmer3* shows a normal localization of OsHUS1. (B) *zep1* displays a normal localization of OsHUS1. (C) OsHUS1 is absent in *pair1*. Bars, 5 µm.
(TIF)

Table S1 Primers used for *OsHUS1* map-based cloning.
(DOCX)
