# Supplementary figures and images for: OsHUS1 Facilitates Accurate Meiotic Recombination in Rice
Source: PLoS Genet. 2014 Jun 5;10(6):e1004405. doi: 10.1371/journal.pgen.1004405 (PMC4046934; doi:10.1371/journal.pgen.1004405)

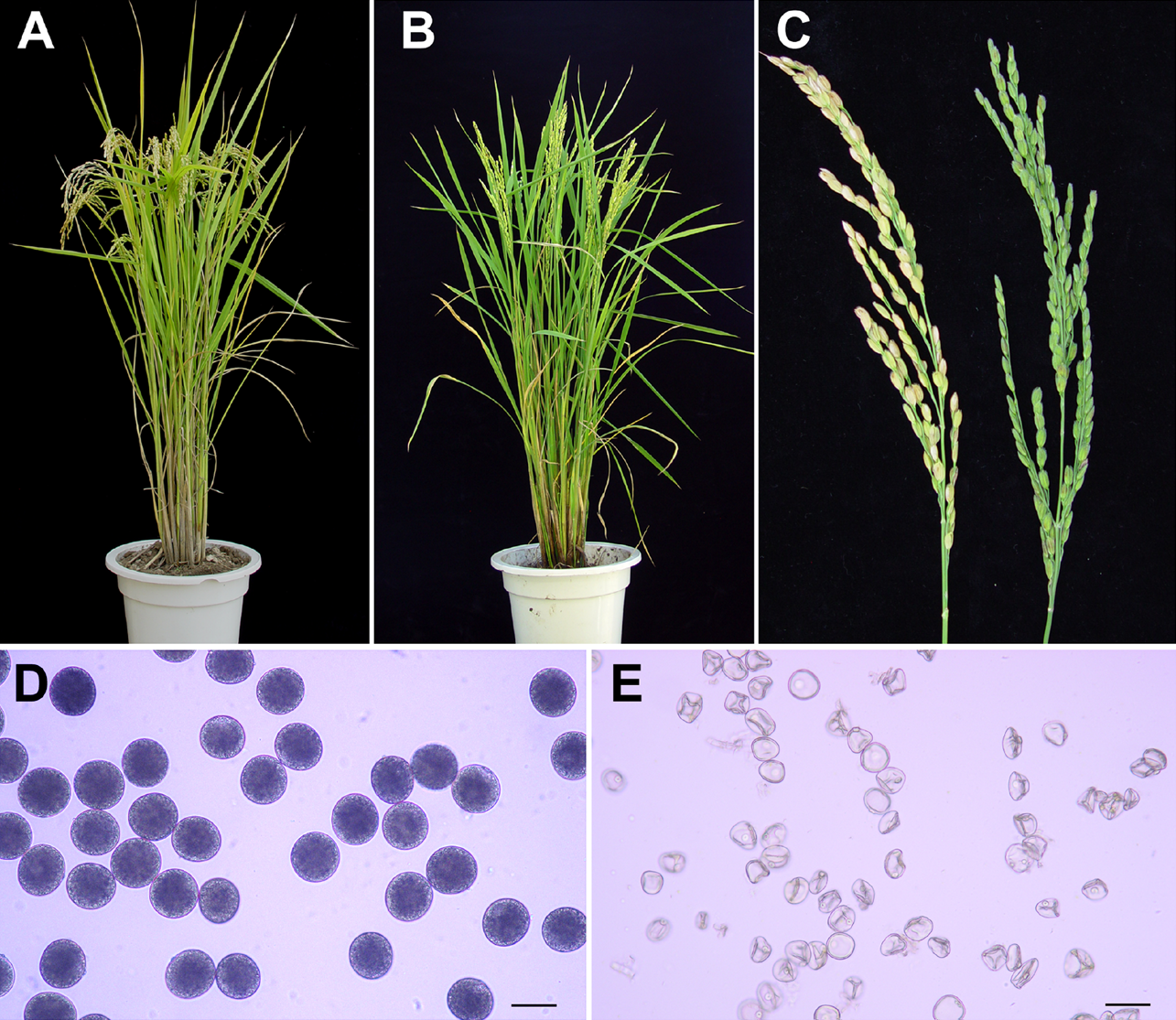

Supplement: Figure S1 — Phenotype of the Oshus1-1 mutant. (A) A wild-type plant; (B) A Oshus1-1 plant; (C) Comparison of a wild-type (left) and a Oshus1-1 panicle (right); (D, E) I2-KI staining of pollen grains in the wild type (D) and Oshus1-1 mutant (E). Bars, 50 µm. (TIF) [file pgen.1004405.s001.tif]

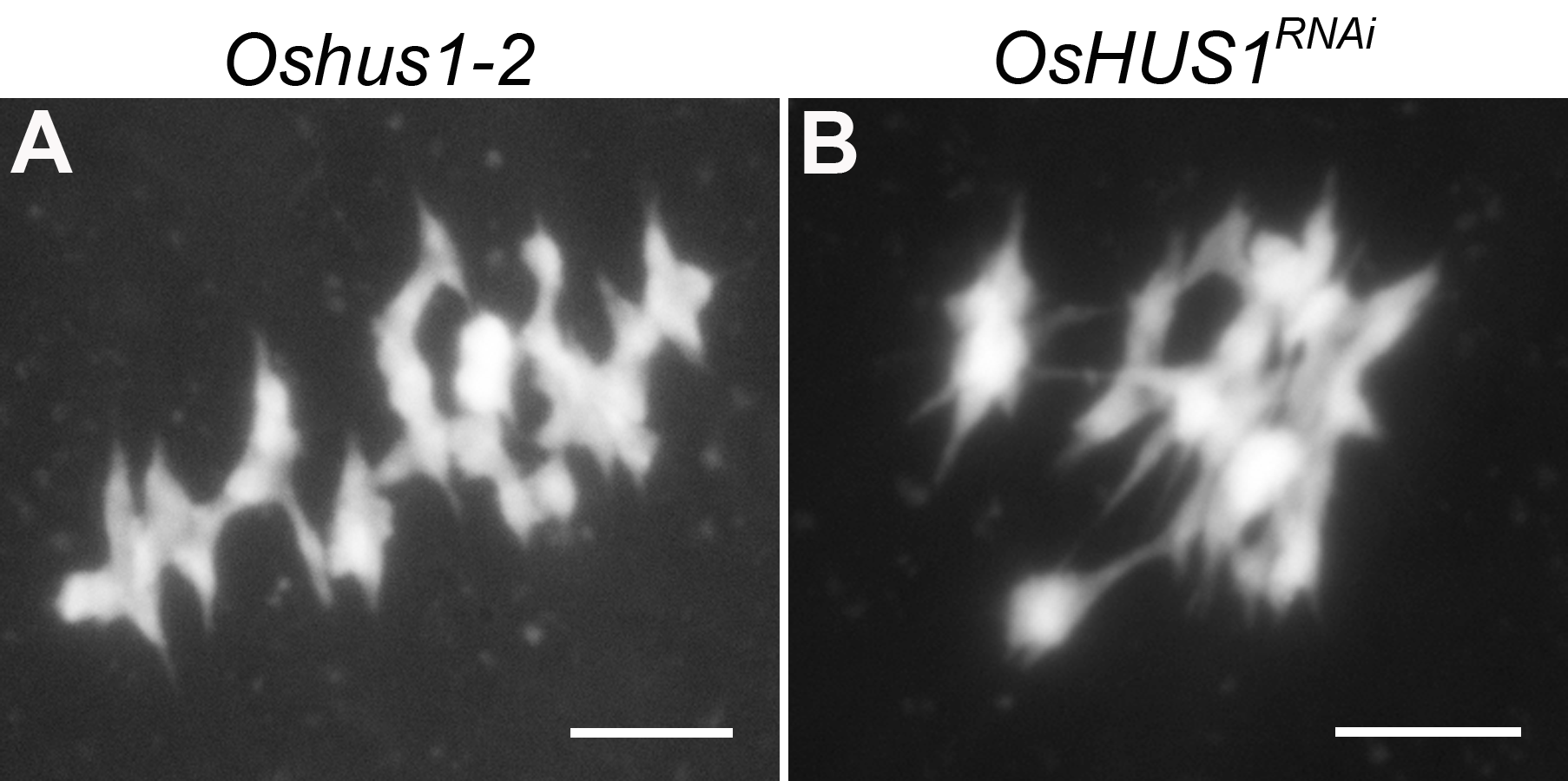

Supplement: Figure S2 — Meiotic chromosomes at Metaphase I in Oshus1-2 and OSHUS1 RNAi plant. (A) Oshus1-2. (B) An OSHUS1 RNAi line. Scale bars, 5 µm. (TIF) [file pgen.1004405.s002.tif]

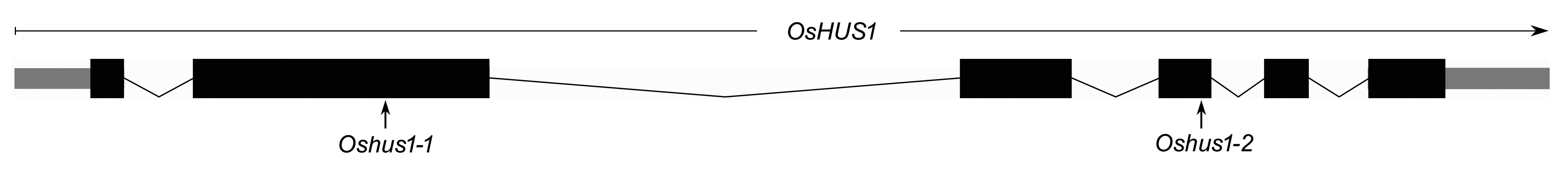

Supplement: Figure S3 — Structure of the OsHUS1 gene. Exons are represented by black boxes. Gray boxes show the untranslated regions. The position of the Oshus1 mutation is indicated by an arrow. (TIF) [file pgen.1004405.s003.tif]

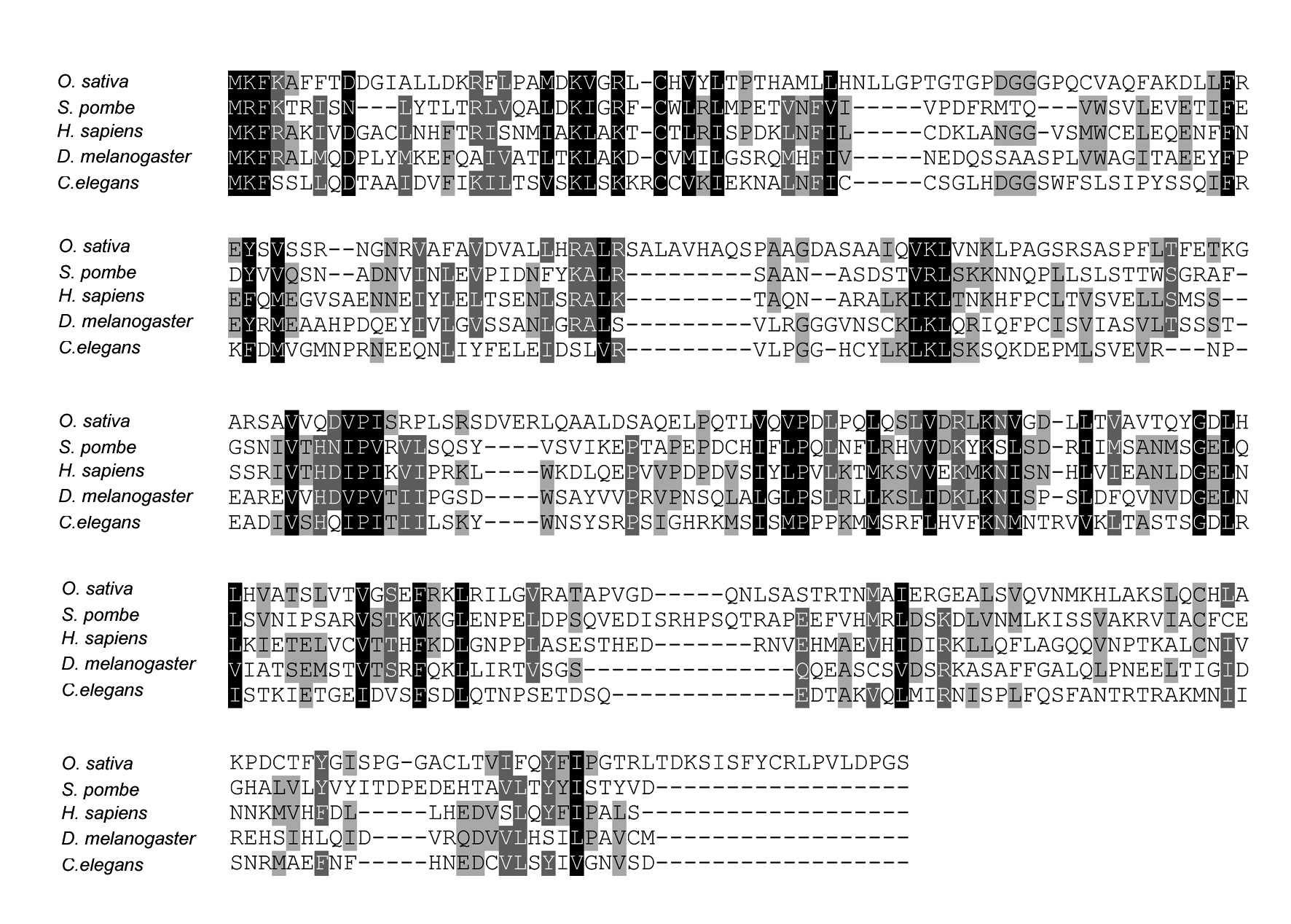

Supplement: Figure S4 — Alignment of HUS1 homologues. Identical amino acids are shaded in black whereas similar amino acids are shaded in gray. (TIF) [file pgen.1004405.s004.tif]

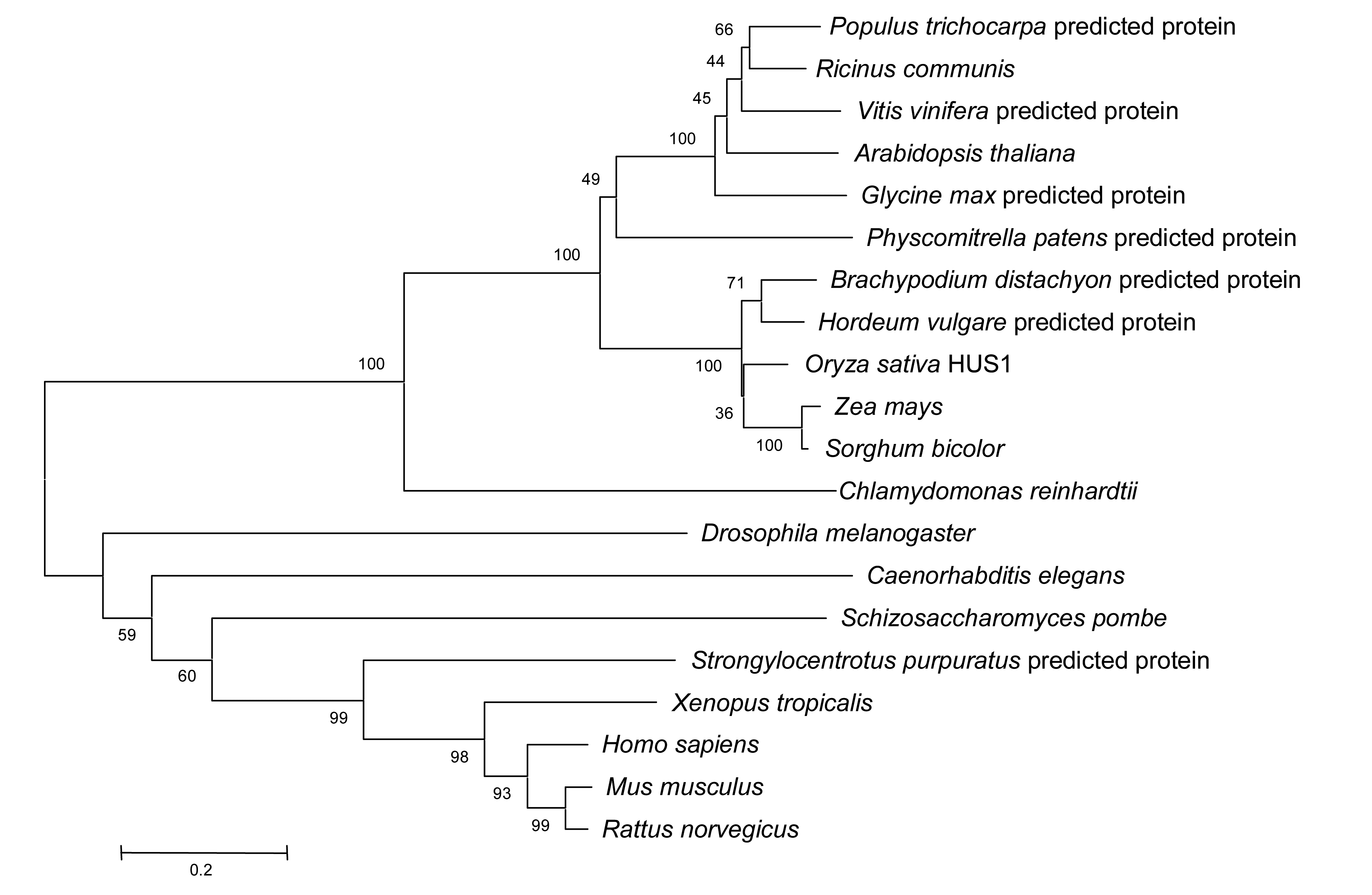

Supplement: Figure S5 — Phylogenetic tree of the 20 homologs defined by OsHUS1. The tree is constructed using MEGA 4.0 based on the neighbor-joining method. Numbers next to branches are clade credibility values. (TIF) [file pgen.1004405.s005.tif]

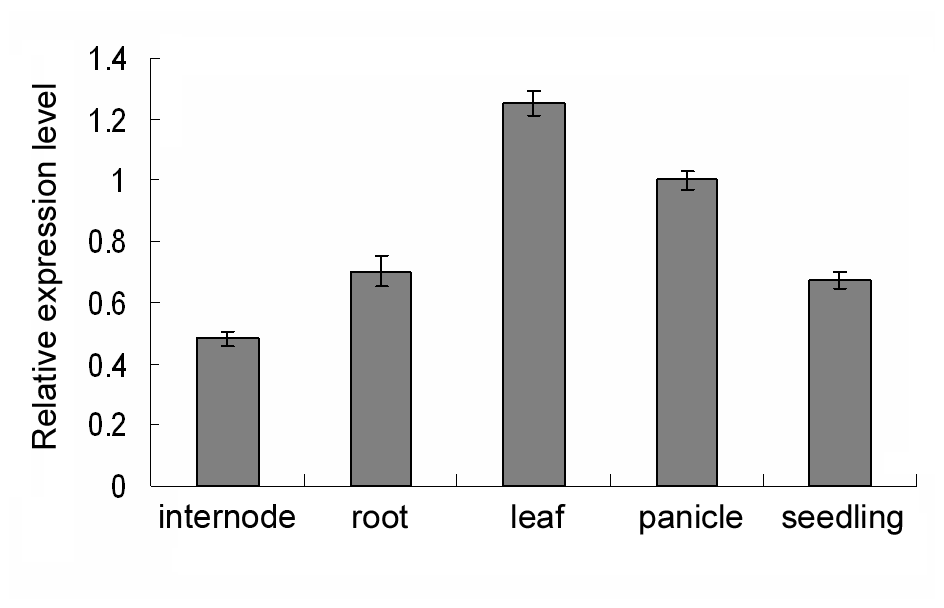

Supplement: Figure S6 — Relative expression level of OsHUS1 in different tissues analyzed by quantitative RT-PCR. Values are means ±SEM (standard error of mean) of three independent experiments and value of panicle is set as 1. (TIF) [file pgen.1004405.s006.tif]

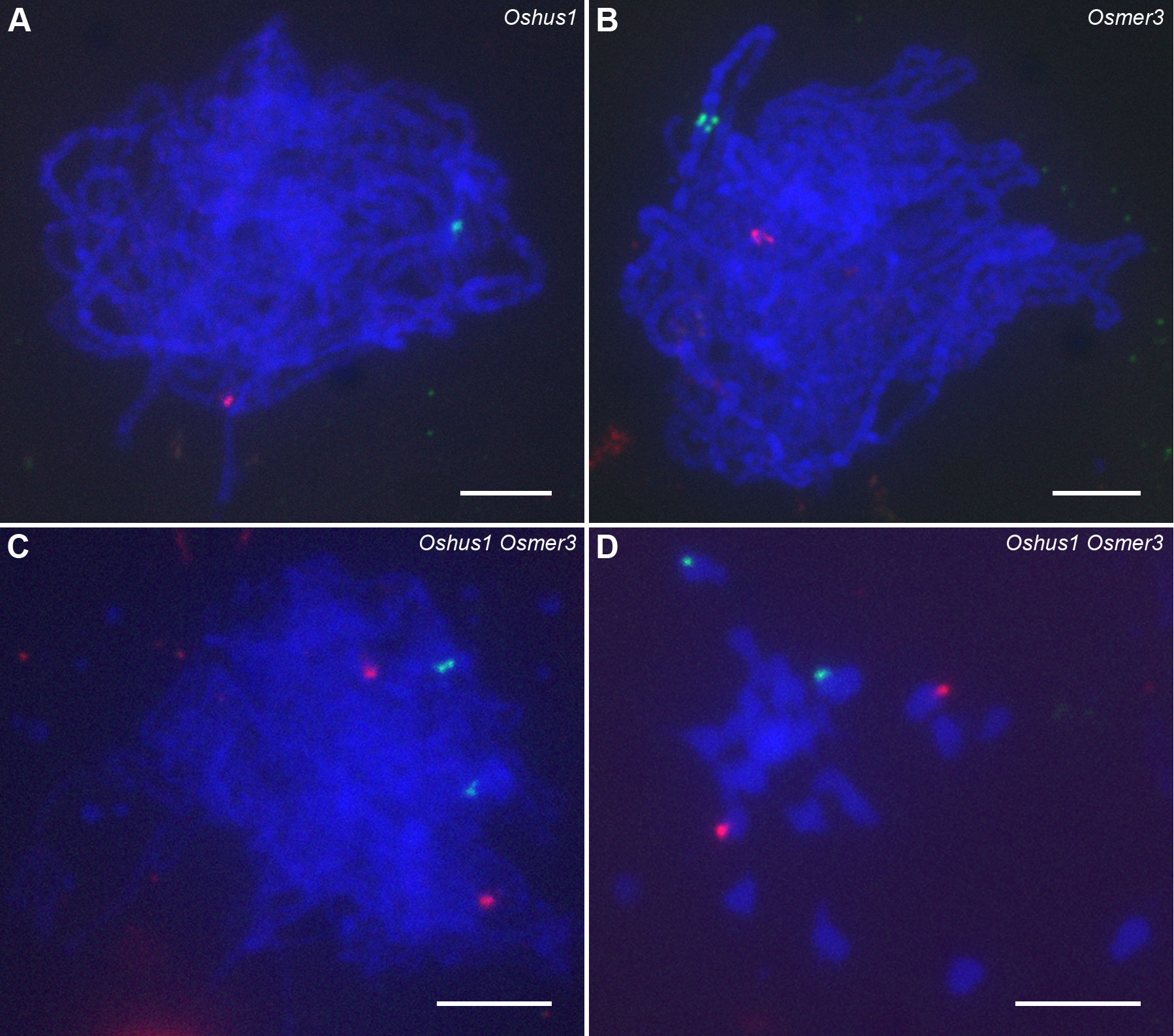

Supplement: Figure S7 — Detection of homologous chromosome pairing revealed by FISH in Oshus1-1, Osmer3 and Osmer3 Oshus1-1. (A–C) Pachytene; (D) Diakinesis; FISH signals of 5S rDNA are in green, signals of the BAC clone a0065A15 on the long arm of chromosome 9 are in red, and chromosomes are in blue stained with DAPI. Bars, 5 µm. (TIF) [file pgen.1004405.s007.tif]

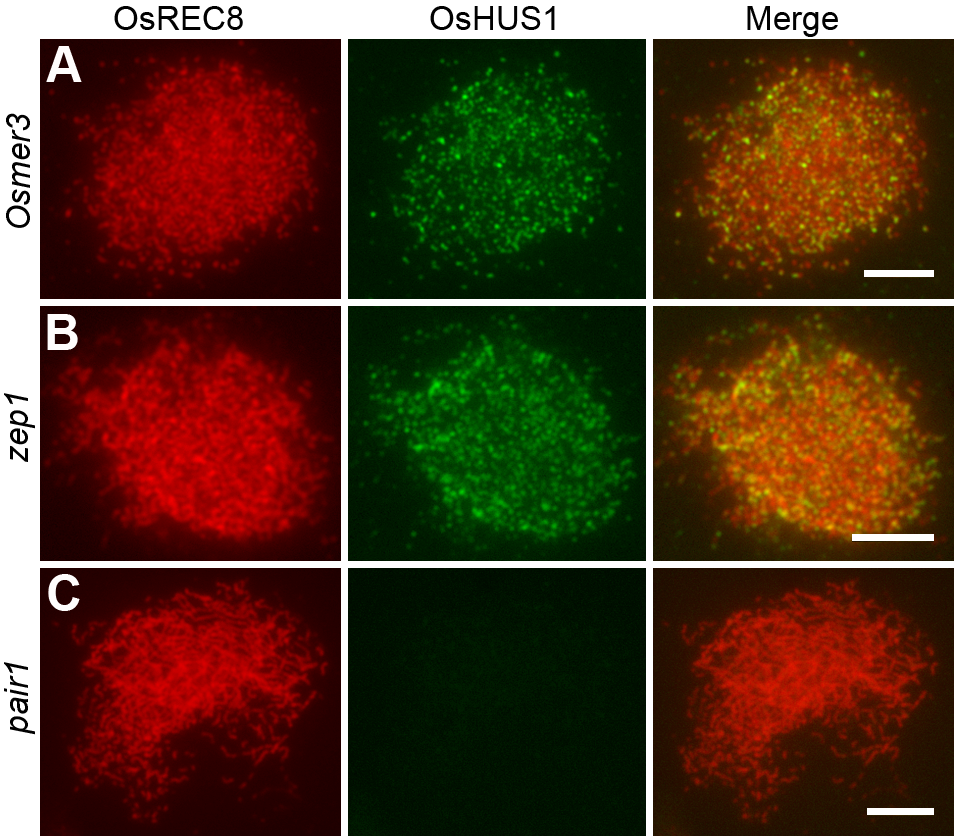

Supplement: Figure S8 — Dual immunolocalization of OsREC8 and OsHUS1 in Osmer3, zep1 and pair1 PMCs. (A) Osmer3 shows a normal localization of OsHUS1. (B) zep1 displays a normal localization of OsHUS1. (C) OsHUS1 is absent in pair1. Bars, 5 µm. (TIF) [file pgen.1004405.s008.tif]
