# Supplementary material for: OsHUS1 Facilitates Accurate Meiotic Recombination in Rice
Source: PLoS Genet. 2014 Jun 5;10(6):e1004405. doi: 10.1371/journal.pgen.1004405 (PMC4046934; doi:10.1371/journal.pgen.1004405)
Supplement: Table S1 — Primers used for OsHUS1 map-based cloning. (DOCX) [file pgen.1004405.s009.docx]

**Table S1** Primers used for *OsHUS1* map-based cloning

|  | Forward | Reverse |
| --- | --- | --- |
| M1 | CATATGATGCATGGAACACC | CTGTACTCGCTTTGAGATGC |
| M2 | AAGGCATCAGAAGCACGG | CATTATTGAGGAGGGGAG |
| M3 | CAGATAGCATCAACTGTC | GAATGTTAATGGAAGCGA |
| M4 | CCCATAGTTTATCTTTTTTAGTA | GTGGTCTAAATAAGCCTTAC |
| M5 | GCTCTATCCCCATTTCTCTA | ATCTACAAATGGGAGACGAC |
